# Associations of epigenetic aging with self-rated health, access to care, and healthcare utilization in a representative sample of United States adults

**DOI:** 10.1186/s13148-025-01887-z

**Published:** 2025-05-14

**Authors:** Jamaji C. Nwanaji-Enwerem, Dennis Khodasevich, Nicole Gladish, Hanyang Shen, Anne K. Bozack, Saher Daredia, Belinda L. Needham, David H. Rehkopf, Andres Cardenas

**Affiliations:** 1 Department of Emergency Medicine, Center for Health Justice, and Center of Excellence in Environmental Toxicology, Perelman School of Medicine, University of Pennsylvania, HUP, Ground Ravdin, 3400 Spruce Street, Philadelphia, PA 19104 USA; 2 Department of Epidemiology and Population Health, Stanford University, Palo Alto, CA USA; 3 Division of Epidemiology, UC Berkeley School of Public Health, Berkeley, CA USA; 4 Department of Epidemiology, Center for Social Epidemiology and Population Health, School of Public Health, University of Michigan, Ann Arbor, MI USA

**Keywords:** DNA methylation age, NHANES, B2M, CRP, Leptin, Packyears

## Abstract

**Background:**

Health status is closely linked to both healthcare access and utilization. While previous research has identified associations between health status and DNA methylation-based biomarkers of aging (epigenetic aging), studies exploring these relationships in the context of healthcare access and utilization remain limited. To address this gap, we analyzed cross-sectional associations in a representative sample of 2,343 U.S. adults from the 1999–2000 and 2001–2002 cycles of the National Health and Nutrition Examination Survey (NHANES). Our study examined the relationships of self-rated health status, healthcare access, and healthcare utilization with seven epigenetic aging biomarkers: HannumAge, HorvathAge, SkinBloodAge, PhenoAge, GrimAge2, DNAm Telomere Length (DNAmTL), and DunedinPoAm.

**Results:**

After adjusting for chronological age, demographics, lifestyle factors, and health insurance, participants with good–excellent self-rated health had a 1.58-year lower PhenoAge (95% CI − 2.54, − 0.62 *P* = 0.006) and a 1.16-year lower GrimAge2 (95% CI − 1.80, − 0.53, *P* = 0.004) than participants with poor-fair health. Participants who reported having a routine place where they received healthcare had a lower GrimAge2 (β = − 1.44-years, 95% CI − 2.66, − 0.22, *P* = 0.03) than participants without a routine healthcare location. Participants with ≥ 10 healthcare visits in the prior year had a shorter DNAmTL (β = − 0.05-kb, 95% CI − 0.09, − 0.01, *P* = 0.02) than participants with < 10 visits. After including additional adjustments for estimated leukocyte proportions, participants who were hospitalized overnight in the prior year had a shorter DNAmTL (β = − 0.05-kb, 95% CI − 0.08, − 0.01, *P* = 0.02) than non-hospitalized individuals.

**Conclusions:**

Our findings reinforce previous reports linking better health status to lower epigenetic aging and provide new evidence of associations of epigenetic aging with measures of healthcare access and utilization. If validated, these findings suggest that epigenetic aging biomarkers may be useful in studying disease processes and assessing health outcomes related to access and utilization.

## Background

Since gaining prominence in 2013 [1, 2], DNA methylation-based biomarkers of aging (also known as epigenetic clocks) have demonstrated robust utility for studying environmental, lifestyle, behavioral, and molecular factors that can impact the aging process [1–7]. Beyond the public health impact of these biomarkers, their robust relationships with morbidity and mortality continues to spur immense interest in their potential usefulness in clinical practice. However, a key question remains: can epigenetic aging measures one day play a role in patient care [8]? Not only will it be important to determine which individual or combination of epigenetic aging biomarkers are clinically useful, but understanding what clinical contexts to apply these biomarkers in is equally important. Disease screening, monitoring of preventative lifestyle habits, risk stratification of patients with disease, and evaluating the impacts of therapeutic interventions are among the clinical contexts with ongoing promise. For screening, evidence suggests that epigenetic age when paired with neuroimaging in healthy adults may help identify individuals at risk for cognitive decline later in life [9]. In the context of preventative lifestyle habits, a trial of 43 healthy adult males randomized to no intervention or an 8-week regimen of probiotics, phytonutrients, and guidance on diet, exercise, relaxation, and sleep demonstrated a 3.23-year lower HorvathAge in the treatment arm [10]. Considering risk stratification in disease, epigenetic age acceleration (PhenoAge and GrimAge) has been associated with time to relapse in chronic lymphocytic leukemia [11]. From the perspective of evaluating therapeutic interventions, women with breast cancer who were treated with chemotherapy demonstrated epigenetic age acceleration (PhenoAge, GrimAge, and DunedinPACE) from their baseline, reflecting the cytotoxicity of this therapy [12].

While the aforementioned research has focused on clinical interventions, the direct relationship of epigenetic aging measures with fundamental healthcare access and utilization patterns remains understudied. Although these relationships represent a public health rather than clinical focus, establishing these baseline associations is critical for: (1) understanding how healthcare disparities may become biologically embedded, and (2) properly contextualizing future clinical applications of epigenetic clocks in healthcare settings. It is well appreciated that access to healthcare is intertwined with health status as access is important for obtaining treatments and general health maintenance [13, 14]. Similarly, healthcare utilization is often related to health status as individuals with serious ailments, chronic disease, and disabilities often need more healthcare services [15, 16]. Although research exists documenting relationships of epigenetic age with objective and self-rated measures of health status [3, 17, 18], evidence of direct relationships with healthcare access and utilization could prompt additional important considerations of these variables as confounders or mediators in future research, including clinical-studies.

In the present study, we analyze data from the National Health and Nutrition Examination Survey (NHANES), a nationally representative sample of United States (U.S.) adults, to investigate the cross-sectional relationship between self-rated health status and epigenetic age. We then examine the relationships of measures of healthcare access and utilization with epigenetic aging. Given the established associations of greater epigenetic aging with increased morbidity and mortality [19], we hypothesize that poorer self-rated health will be associated with greater epigenetic aging. Similarly, under the premise that limited healthcare access and higher healthcare utilization are often associated with worse health status, we also hypothesize that both will be associated with greater epigenetic aging.

## Methods

### Study population

The National Center for Health Statistics (NCHS) assesses the health of the noninstitutionalized U.S. population through NHANES interviews, physical examinations, and laboratory tests. In this study, we examined the relationships of self-rated health, healthcare access, and healthcare utilization with epigenetic age using publicly available data from the 1999–2000 and 2001–2002 NHANES cycles. Our sample included 2,532 adults aged 50 years and older. To protect participant privacy, NHANES top-coded the ages of individuals 85 years and older as 85 years (n = 130), making their exact chronological ages unknown. We excluded these participants to prevent misclassification errors in epigenetic age measures. We also removed individuals whose DNAm-predicted sex did not match their self-reported sex (n = 56), resulting in a final sample of 2,346 participants. Of these, 2,343 had data on self-rated health status (n = 3 missing). All participants provided written informed consent, and the NCHS Research Ethics Review Board approved the study protocols (protocol #98–12).

### Self-rated health

As part of the “Hospital Utilization & Access to Care” questionnaire [20, 21], participants provided information on self-rated health by answering the question, “Would you say your health in general is …” with possible responses of “Excellent”, “Very good”, “Good”, “Fair” or “Poor.” Participants who declined to respond, were unsure of their health status, or had missing data were excluded from the analyses. To increase statistical power for comparisons across groups, we dichotomized self-rated health into two categories: “Good–Excellent” health and “Poor-Fair” health.

Although our analysis is based on cross-sectional data, we considered changes in health status over time by including a measure of self-reported health compared to the previous year. Participants were asked, “Compared with 12 months ago, would you say your health is now…” with response options: “Better”, “Worse”, or “About the same.” Those who declined to respond, were unsure of their health status, or had missing data were excluded from the analyses.

### Healthcare access

Our analysis included two measures of healthcare access available in both 1999–2000 and 2001–2002 NHANES cycles. First, participants were asked, “Is there a place that you usually go when you are sick or need advice about your health?” Responses were coded as “Yes” if participants responded “Yes” or “There is more than one place.” Responses were coded as “No” if they responded, “There is no place.” Subsequently, participants were asked, “What kind of place do you go to most often: is it a clinic, doctor’s office, emergency room, or some other place?” Responses were, “Clinic or health center,” “Doctor’s office or HMO,” “Hospital emergency room,” “Hospital outpatient department,” or “Some other place.” For this variable, “hospital emergency room” was set as the reference group because emergency departments are never closed and cannot deny treatment based on a patient’s ability to pay [22]. For both healthcare access measures, participants who declined to respond, were unsure of their response, or had missing data were excluded from the analyses.

### Healthcare utilization

We included two measures of healthcare utilization available in both 1999–2000 and 2001–2002 NHANES cycles. First, participants were asked, “During the past 12 months, how many times have you seen a doctor or other health professional about your health at a doctor’s office, a clinic, hospital emergency room, at home or some other place? Do not include times you were hospitalized overnight.” Responses included “None”, “1,” “2 to 3,” “4 to 9,” “10 to 12,” “13 or more.” After analyzing all participant answers, “4 to 9” was the median, so we dichotomized the variable as “ < 10” and “ ≥ 10 visits” as a way of designating high visit utilizers. Second, participants were asked, “During the past 12 months, were you a patient in a hospital overnight? Do not include an overnight stay in the emergency room.” Responses were coded as “Yes” or “No.” For all healthcare utilization measures, participants with missing data, uncertain responses, or who declined to answer were excluded from the analyses.

### DNA methylation and epigenetic age

We obtained epigenetic age measures and DNA methylation-based leukocyte proportion estimates from the NHANES website (https://wwwn.cdc.gov/nchs/nhanes/dnam/), which also provides detailed information on DNA methylation analysis and processing. Briefly, DNA was extracted from whole blood samples collected from NHANES participants aged 50 years and older during the 1999–2000 and 2001–2002 cycles. Genome-wide DNA methylation was then assessed using the Illumina EPIC BeadChip array.

Our study included seven epigenetic age measures: HannumAge, HorvathAge, SkinBloodAge, PhenoAge, GrimAge2, DunedinPoAm, and DNA methylation-based Telomere Length (DNAmTL). These measures were selected a priori based on their well-established associations with health outcomes [1–6, 23, 24]. The HannumAge, HorvathAge, and SkinBloodAge measures primarily predict chronological age based on DNA methylation patterns, although research has linked them to broader health indicators [1, 2, 24, 25]. PhenoAge, a leading biomarker of healthspan, was developed using a composite measure of nine clinical variables: albumin, creatinine, glucose, C-reactive protein, lymphocyte percentage, mean cell volume, red cell distribution width, alkaline phosphatase, and white blood cell count [6]. GrimAge2, a lifespan biomarker, integrates chronological age, gender, and ten DNA methylation surrogates for cigarette pack-years and plasma protein markers, including adrenomedullin (ADM), beta-2-microglobulin (B2M), C-reactive protein (CRP), cystatin C, growth differentiation factor-15 (GDF-15), hemoglobin A1c (A1c), leptin, plasminogen activator inhibitor-1 (PAI1), and tissue inhibitor metalloproteinase-1 (TIMP1) [4]. DNAmTL estimates telomere length based on DNA methylation patterns [5]. DunedinPoAm measures the pace of biological aging by assessing morbidity-related biomarkers. This metric was developed by analyzing longitudinal changes in 18 organ function biomarkers among individuals of the same chronological age, offering a robust indicator of aging pace [3]. As our analysis utilized epigenetic aging measures publicly available in NHANES, we could not include more recent clocks like DunedinPACE, which were not available at the time of this study.

### Statistical analysis

We used the R ‘Survey’ package to perform generalized linear regression models, incorporating NHANES-provided participant sample weights designed for the epigenetic clock subsample [26]. To examine the associations between self-rated health, healthcare access, and healthcare utilization with each epigenetic age measure, we applied the *svyglm* function in R, which accounts for the survey’s complex design. Our main model covariates were determined a priori and included chronological age (continuous, in years) and its quadratic term, sex (female vs. male), and self-identified ethnicity/race (Non-Hispanic White, Mexican American, Other Hispanic, Non-Hispanic Black, Other Race). We also adjusted for health insurance (yes vs. no), education level (less than high school, high school diploma/GED, more than high school), occupation (white-collar/professional, white-collar/semi-routine, blue-collar/high-skill, blue-collar/semi-routine, or no work), and poverty-to-income ratio (continuous), alcohol intake (abstainer, moderate drinker, heavy drinker), body mass index (BMI [kg/m^2^]; continuous), smoking status (never, former, current), and physical activity (moderate/vigorous activity in the last 30 days: yes vs. no). Health access and utilization models also included self-rated health status as a covariate. When self-rated health, healthcare access, and healthcare utilization were associated with GrimAge2, we used the same covariate adjustments in models examining associations with DNAm-predicted blood biomarker components of GrimAge2 to test drivers of associations. To address missing covariate data, we used multiple imputation via the *MICE* function in R, generating 10 imputed datasets. The estimates from these datasets were then pooled using the *pool* function in R [27]. Because healthcare utilization is strongly correlated with chronological aging, we conducted a secondary analysis where we employed the same modeling framework—omitting adjustments for chronological age—to compare associations of self-rated health, healthcare access, and healthcare utilization with chronological age versus those observed with epigenetic age biomarkers.

We performed five sensitivity analyses. To assess the impact of leukocyte proportions on our results, the first sensitivity analysis involved models that included additional adjustments for estimated leukocyte proportions (B cells, CD4 cells, CD8 cells, NK cells, monocytes, and neutrophils). To evaluate the impact of health insurance on our results, the second sensitivity analysis involved models that did not include health insurance as a covariate. Given that health status has strong relationships with healthcare access and utilization, the third sensitivity analysis involved healthcare access and utilization models that did not include self-rated health status as a covariate. To assess the impact of dichotomization of self-rated health status on our analysis, we conducted a fourth sensitivity analysis exploring associations of self-rated health status in its discrete categories with epigenetic aging. Finally, given previously reported differences in directly measured telomere length versus DNAmTL [28], for measures associated with DNAmTL, we applied the same covariate framework to examine their relationships with directly measured leukocyte telomere length available in NHANES. Directly measured telomere length was quantified from whole blood DNA using quantitative PCR, which determines the relative telomere repeat copy number to single-copy gene copy number (T/S ratio) [29]. The T/S ratio was then converted to kilobase (kb) pairs using the formula: 3,274 + 2,413 × (T/S)/1,000 [30]. These methods have been previously described [31]. All statistical analyses were conducted using R Version 4.4.1 (R Core Team, Vienna, Austria). To account for multiple comparisons across seven independent epigenetic clocks, statistical significance was set at a Bonferroni-adjusted p-value of < 0.007 (0.05/7). P-values < 0.05 were considered marginal.

## Results

### Study sample characteristics

**Fig. 1:**
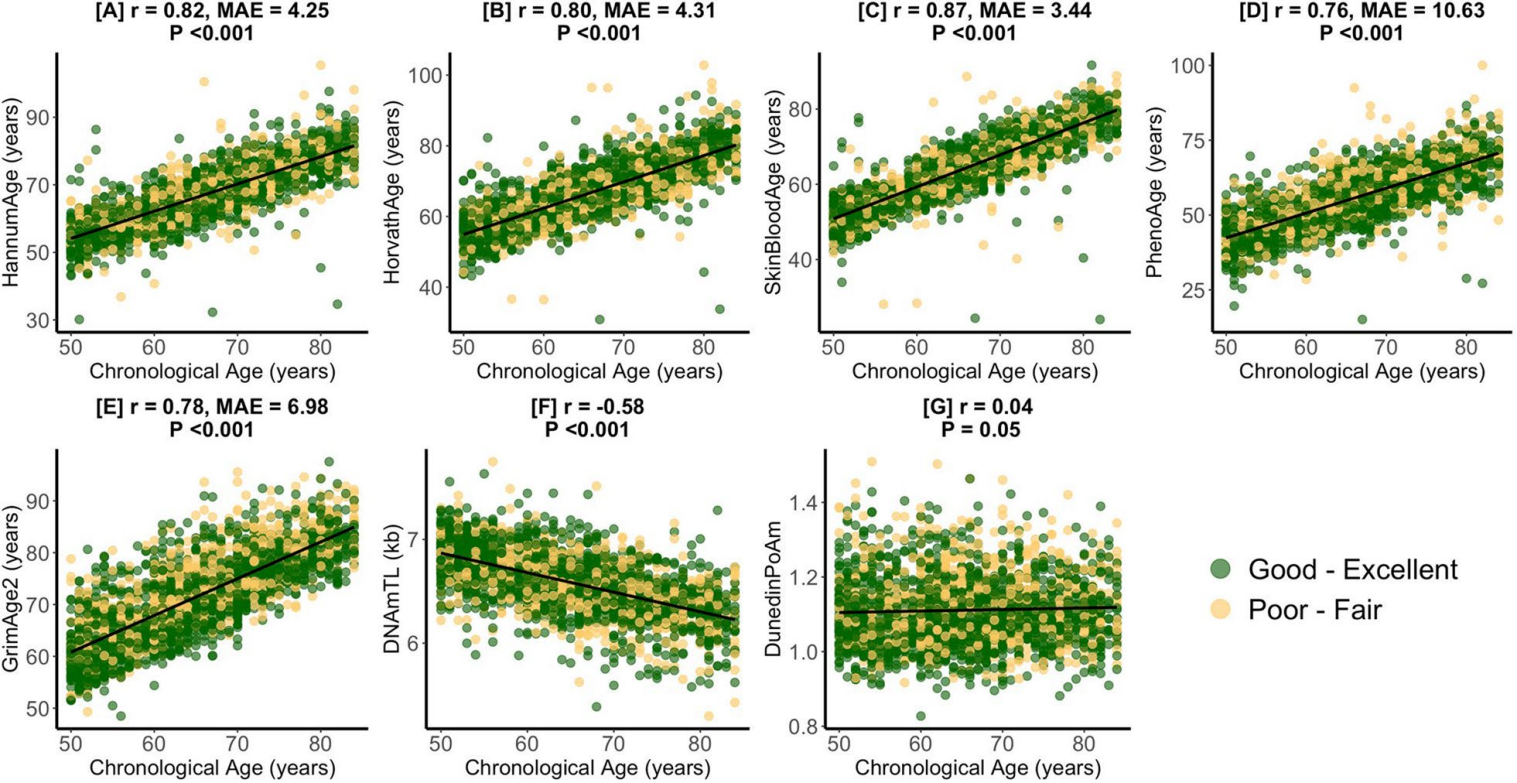
Pearson Correlations (r) and Median Absolute Error (MAE) of Epigenetic Age with Chronological Age. Presents the chronological age and epigenetic age correlation coefficients and median absolute errors for the study sample (n = 2343) for HannumAge (**A**), HorvathAge (**B**), SkinBloodAge (**C**), PhenoAge (**D**), GrimAge2 (**E**), DNAmTL (**F**), and DunedinPoAm (**G**). Values for participants with good–excellent and poor–fair self-rated health are in green and yellow respectively

Figure S1 presents a flow chart describing how the final study sample was achieved. Table 1 presents the study sample characteristics prior to the application of survey weights. Participants had a mean (sd) chronological age of 65.1 (9.3) years. Most participants had at least a high school diploma (55%), were blue-collar workers (52%), and were male (51%). 39% of participants were non-Hispanic White. With respect to health status and utilization, most participants reported having good–excellent health (67%), the same health as compared to last year (71%), having a routine place where they receive healthcare (92%), and that their routine place for healthcare was not the emergency department (91%). Few participants saw a doctor ≥ 10 times last year (17%) or had an overnight hospital stay in the last year (15%). With respect to health behaviors, most patients consumed alcohol (52%), were not physically active (51%), and reported having health insurance (88%). 45% of participants were never smokers. Figure 1 describes strong correlations of chronological age with epigenetic biomarkers in study sample. DNAmTL was negatively correlated with chronological age (r = − 0.58, *P* < 0.001) while SkinBloodAge (r = 0.87, Median Absolute Error [MAE] = 3.44-years, *P* < 0.001) had the strongest positive correlation with chronological age.

**Table 1:** Unweighted Summary Statistics Among U.S. Adults from NHANES 1999–2002 (n = 2343)

Demographic variables	
Chronological Age (years), mean (sd)	65.1 (9.3)
Epigenetic Age/Clocks, mean (sd)	
HannumAge (years)	66.3 (9.2)
HorvathAge (years)	66.1 (8.6)
SkinBloodAge (years)	63.6 (9.1)
PhenoAge (years)	54.9 (10.1)
GrimAge2 (years)	71.5 (8.4)
DNAm Telomere Length (TL) (kb)	6.6 (0.3)
DunedinPoAm	1.1 (0.1)
Education, n (%)	
Less Than High School	1060 (45)
High School Diploma (including GED)	487 (21)
More Than High School	794 (34)
Missing	2 (0)
Occupation, n (%)	
Blue-collar (high skill)	312 (13)
Blue-collar (semi-routine)	919 (39)
White-collar (high skill)	520 (22)
White-collar (semi-routine)	396 (17)
Never worked	60 (3)
Missing	136 (6)
Poverty to Income Ratio, mean (sd)	
	2.6 (1.6)
Missing	267
Race/Ethnicity Category, n (%)	
Mexican American	680 (29)
Other Hispanic	151 (7)
Non-Hispanic White	921 (39)
Non-Hispanic Black	511 (22)
Other Race	80 (3)
Sex, n (%)	
Male	1201 (51)
Female	1142 (49)
Health Status, Access, and Utilization Variables	
Health Status, n (%)	
Good–Excellent	1559 (67)
Poor-Fair	784 (33)
Health Compared to Last Year, n (%)	
Better	370 (16)
Same	1667 (71)
Worse	305 (13)
Missing	1 (0)
Has Routine Place for Healthcare, n (%)	
Yes	2164 (92)
No	179 (8)
Routine Place is not the Emergency Department, n (%)	
Yes	2126 (91)
No	36 (1)
Missing	181 (8)
≥ 10 Visits in the Last Year, n (%)	
Yes	395 (17)
No	1947 (83)
Missing	1 (0)
Overnight Hospital Patient in the Last Year, n (%)	
Yes	342 (15)
No	2000 (85)
Missing	1 (0)
Health Behavior Variables	
Alcohol Intake, n (%)	
Abstainer	1008 (43)
Moderate Drinker	1135 (48)
Heavy Drinker	84 (4)
Missing	116 (5)
Body Mass Index (kg/m^2^), mean (sd)	
	28.8 (5.8)
Missing	83
Health Insurance, n (%)	
Yes	2047 (88)
No	265 (11)
Missing	31 (1)
Smoking, n (%)	
Current	373 (16)
Former	903 (39)
Never	1062 (45)
Missing	5 (0)
Physically Active, n (%)	
Yes	1145 (49)
No	1196 (51)
Missing	2 (0)

Table S1 presents unweighted relationships of healthcare access and utilization variables by self-rated health status. A greater proportion of participants with poor-fair health had worse health compared to the past year than was observed in participants with good–excellent health (24% vs. 7%, *P* < 0.001). When compared to their counterparts with good–excellent health, a higher proportion of poor-fair health participants had ≥ 10 visits in the last year (28% vs 11%, *P* < 0.001) and had an overnight hospitalization in the last year (23% vs 10%, *P* < 0.001). We observed no statistically significant relationship of having a routine place for healthcare with self-rated health.

### Relationships of health status with epigenetic age

Table 2 describes adjusted associations of self-rated health status with epigenetic aging. In participants reporting good–excellent health, a significantly lower PhenoAge (β = − 1.58-years, 95% CI − 2.54, − 0.62, *P* = 0.006), GrimAge2 (β = − 1.16-years, 95% CI − 1.80, -0.53, *P* = 0.004), and DunedinPoAm (β = − 0.02, 95% CI − 0.03, − 0.003, *P* = 0.02) were observed compared to participants reporting poor-fair health. In the sensitivity analysis, the PhenoAge (β = − 1.15-years, 95% CI − 1.95, − 0.34, *P* = 0.01) and GrimAge2 (β = − 0.82-years, 95% CI − 1.38, − 0.26, *P* = 0.01) relationships were slightly attenuated but remained significant after adjusting for estimated leukocyte proportions. Participants reporting good–excellent health had lower GrimAge2 cigarette packyears (β = − 1.54, 95% CI − 3.04, − 0.04, *P* = 0.045) and lower levels of GrimAge2 components B2M (β = − 14933.17, 95% CI − 28777.27, − 1089.07, *P* = 0.04) before adjusting for leukocyte proportions and lower levels of CRP (β = − 0.08, 95% CI − 0.15, − 0.01, *P* = 0.04) and leptin (β = − 359.08, 95% CI − 692.38, − 25.78, *P* = 0.04) after adjusting for leukocyte proportions compared to individuals reporting poor-fair health. We observed similar relationships in models that were not adjusted for health insurance (Table S2). Similar trends were also observed with more granular categories of self-rated health status (Table S3). We did not observe any statistically significant relationships of self-rated health compared to the prior year with any epigenetic age measure (Table S4).

**Table 2:** Adjusted associations of Self-Rated Health Status with Epigenetic Aging Biomarkers (n = 2343)^†^

	Main model		Leukocyte-adjusted model	
Biomarker	Estimate (95% CI)	*P*-value	Estimate (95% CI)	*P*-value
HannumAge (years)	− 0.58 (− 1.65, 0.48)	0.23	− 0.30 (− 1.35, 0.76)	0.52
HorvathAge (years)	− 0.20 (− 1.08, 0.68)	0.60	− 0.07 (− 0.91, 0.77)	0.85
SkinBloodAge (years)	− 0.26 (− 1.01, 0.50)	0.44	− 0.13 (− 0.93, 0.67)	0.71
PhenoAge (years)	− 1.58 (− 2.54, − 0.62)	0.006	− 1.15 (− 1.95, − 0.34)	0.01
GrimAge2 (years)	− 1.16 (− 1.80, − 0.53)	0.004	− 0.82 (− 1.38, − 0.26)	0.01
DNAmTL (kb)	0.01 (− 0.03, 0.05)	0.52	0.01 (− 0.02, 0.05)	0.41
DunedinPoAm	− 0.02 (− 0.03, − 0.003)	0.02	− 0.01 (− 0.02, 0.0004)	0.06
GrimAge2 components				
A1c	− 0.005 (− 0.01, 0.0005)	0.07	− 0.005 (− 0.01, 0.0005)	0.07
ADM	− 2.56 (− 5.59, 0.46)	0.08	− 1.97 (− 4.82, 0.87)	0.14
B2M	− 14,933.17 (− 28777.27, − 1089.07)	0.04	− 10261.59 (− 24409.55, 3886.36)	0.13
CRP	− 0.10 (− 0.18, − 0.01)	0.03	− 0.08 (− 0.15, − 0.01)	0.04
Cystatin C	− 3677.57 (− 7726.6, 371.46)	0.07	− 2388.38 (− 5303.76, 527.01)	0.09
GDF15	− 9.14 (− 25.59, 7.32)	0.23	− 6.41 (− 24.24, 11.43)	0.42
Leptin	− 221.52 (− 493.07, 50.03)	0.09	− 359.08 (− 692.38, − 25.78)	0.04
Packyears	− 1.54 (− 3.04, − 0.04)	0.045	− 1.26 (− 2.75, 0.23)	0.08
PAI1	− 320.81 (− 778.98, 137.36)	0.14	− 279.61 (− 703.69, 144.48)	0.16
TIMP1	− 90.77 (− 189.84, 8.3)	0.07	− 37.81 (− 122.8, 47.19)	0.32

Model estimates are for participants with good–excellent self-rated health status (participants with poor–fair self-rated health status serve as the reference group)

^†^Models adjusted for chronological age, chronological age^2^, sex, race/ethnicity, alcohol intake, BMI, education, occupation, physical activity, PIR, health insurance, and smoking. Models with additional adjustments for estimated leukocyte proportions are noted

*P* < 0.007: Bonferroni significant

*P* < 0.05: marginally significant

### Relationships of access to care and utilization with epigenetic age

Table 3 presents the results of models examining the relationships of access to healthcare and utilization with epigenetic age. Participants who reported having a routine place where they received healthcare had marginally lower GrimAge2 levels (β = − 1.44, 95% CI − 2.66, − 0.22, *P* = 0.03) when compared to participants without a routine place for healthcare. Furthermore, marginally lower levels of the epigenetic estimates for CRP (β = − 0.13, 95% CI − 0.26, − 0.01, *P* = 0.04) and leptin (β = − 415.41, 95% CI − 784.04, − 46.78, *P* = 0.03) were observed in participants with a routine place for healthcare (Table S5). Compared to their counterparts with less healthcare visits per year, participants with ≥ 10 visits had marginally lower DNAmTL levels (β = − 0.05, 95% CI − 0.09, − 0.01, *P* = 0.02). Furthermore, only after adjusting for leukocytes, participants who were hospitalized overnight in the last year had marginally lower DNAmTL levels (β = − 0.05, 95% CI − 0.08, − 0.01, *P* = 0.02) when compared to their counterparts who did not have an overnight hospitalization. We observed similar trends in models that were not adjusted for self-rated health status (Table 3) and not adjusted for health insurance (Table S6).

**Table 3:** Adjusted Associations of Healthcare Access and Utilization with Epigenetic Aging Biomarkers^†^

		Main model		Not adjusted for self-rated health model		Leukocyte-adjusted model		
Utilization metric	Biomarker	Estimate (95% CI)	*P*-value	Estimate (95% CI)	*P*-value	Estimate (95% CI)	*P*-value	n
Has Routine Place	HannumAge (years)	− 0.26 (− 1.87, 1.35)	0.70	− 0.25 (− 1.82, 1.32)	0.72	− 0.39 (− 1.71, 0.92)	0.49	2343
Has Routine Place	HorvathAge (years)	− 0.19 (− 1.53, 1.14)	0.73	− 0.19 (− 1.48, 1.1)	0.74	− 0.27 (− 1.59, 1.04)	0.62	2343
Has Routine Place	SkinBloodAge (years)	− 0.20 (− 1.28, 0.89)	0.67	− 0.19 (− 1.23, 0.85)	0.68	− 0.26 (− 1.33, 0.82)	0.57	2343
Has Routine Place	PhenoAge (years)	− 0.33 (− 2.99, 2.34)	0.77	− 0.29 (− 2.95, 2.38)	0.80	− 0.36 (− 2.45, 1.73)	0.68	2343
Has Routine Place	GrimAge2 (years)	− 1.44 (− 2.66, − 0.22)	***0.03***	− 1.41 (− 2.61, − 0.22)	***0.03***	− 1.33 (− 2.66, 0.01)	0.05	2343
Has Routine Place	DNAmTL (kb)	0.03 (− 0.06, 0.12)	0.40	0.03 (− 0.05, 0.12)	0.40	0.03 (− 0.05, 0.1)	0.39	2343
Has Routine Place	DunedinPoAm	− 0.02 (− 0.04, 0.004)	0.09	− 0.02 (− 0.04, 0.004)	0.09	− 0.02 (− 0.04, 0.01)	0.19	2343
Routine Place Not ED	HannumAge (years)	− 0.34 (− 1.89, 1.21)	0.60	− 0.28 (− 1.72, 1.16)	0.66	0.05 (− 1.24, 1.34)	0.93	2162
Routine Place Not ED	HorvathAge (years)	− 0.06 (− 1.39, 1.26)	0.91	− 0.05 (− 1.31, 1.22)	0.93	0.3 (− 1.36, 1.97)	0.67	2162
Routine Place Not ED	SkinBloodAge (years)	− 0.42 (− 2.03, 1.2)	0.54	− 0.38 (− 1.95, 1.18)	0.58	− 0.03 (− 2.08, 2.02)	0.98	2162
Routine Place Not ED	PhenoAge (years)	− 0.13 (− 3.66, 3.41)	0.93	0.07 (− 3.30, 3.44)	0.96	0.08 (− 3.34, 3.5)	0.96	2162
Routine Place Not ED	GrimAge2 (years)	0.27 (− 1.72, 2.25)	0.75	0.42 (− 1.39, 2.23)	0.60	0.43 (− 1.35, 2.21)	0.57	2162
Routine Place Not ED	DNAmTL (kb)	0.05 (− 0.07, 0.16)	0.38	0.04 (− 0.07, 0.16)	0.39	0.01 (− 0.08, 0.11)	0.73	2162
Routine Place Not ED	DunedinPoAm	0.0002 (− 0.04, 0.04)	0.99	0.002 (− 0.03, 0.04)	0.88	0.001 (− 0.03, 0.03)	0.94	2162
≥ 10 Visits Last Yr	HannumAge (years)	0.45 (− 0.69, 1.59)	0.36	0.56 (− 0.55, 1.66)	0.27	0.06 (− 0.96, 1.09)	0.88	2342
≥ 10 Visits Last Yr	HorvathAge (years)	0.44 (− 0.68, 1.57)	0.37	0.47 (− 0.66, 1.59)	0.35	0.11 (− 0.97, 1.19)	0.81	2342
≥ 10 Visits Last Yr	SkinBloodAge (years)	0.26 (− 0.78, 1.3)	0.56	0.30 (− 0.72, 1.32)	0.50	− 0.12 (− 1.11, 0.87)	0.77	2342
≥ 10 Visits Last Yr	PhenoAge (years)	0.24 (− 1.01, 1.48)	0.66	0.55 (− 0.67, 1.78)	0.32	− 0.05 (− 1.22, 1.11)	0.92	2342
≥ 10 Visits Last Yr	GrimAge2 (years)	0.36 (− 0.4, 1.11)	0.29	0.58 (− 0.12, 1.29)	0.09	0.18 (− 0.78, 1.14)	0.66	2342
≥ 10 Visits Last Yr	DNAmTL (kb)	− 0.05 (− 0.09, − 0.01)	***0.02***	− 0.05 (− 0.09, − 0.01)	***0.02***	− 0.03 (− 0.07, 0.003)	0.06	2342
≥ 10 Visits Last Yr	DunedinPoAm	0.01 (− 0.01, 0.02)	0.38	0.01 (− 0.005, 0.02)	0.16	0.01 (− 0.01, 0.02)	0.22	2342
Hospitalized Last Yr	HannumAge (years)	0.26 (− 1.12, 1.64)	0.65	0.37 (− 1.01, 1.76)	0.54	0.64 (− 0.52, 1.81)	0.22	2342
Hospitalized Last Yr	HorvathAge (years)	0.65 (− 0.48, 1.78)	0.21	0.67 (− 0.47, 1.81)	0.20	0.96 (− 0.21, 2.13)	0.09	2342
Hospitalized Last Yr	SkinBloodAge (years)	− 0.08 (− 1.06, 0.91)	0.85	− 0.02 (− 1.00, 0.96)	0.96	0.26 (− 0.84, 1.36)	0.58	2342
Hospitalized Last Yr	PhenoAge (years)	− 0.29 (− 1.63, 1.05)	0.62	0.04 (− 1.34, 1.43)	0.94	0.2 (− 1.22, 1.61)	0.74	2342
Hospitalized Last Yr	GrimAge2 (years)	0.28 (− 0.64, 1.21)	0.48	0.51 (− 0.42, 1.44)	0.23	0.68 (− 0.20, 1.56)	0.11	2342
Hospitalized Last Yr	DNAmTL (kb)	− 0.04 (− 0.08, 0.01)	0.07	− 0.04 (− 0.08, 0.004)	0.07	− 0.05 (− 0.08, − 0.01)	***0.02***	2342
Hospitalized Last Yr	DunedinPoAm	0.01 (− 0.01, 0.03)	0.35	0.01 (− 0.01, 0.03)	0.21	0.01 (− 0.01, 0.03)	0.15	2342

^†^Models adjusted for chronological age, chronological age^2^, sex, race/ethnicity, self-rated health, alcohol intake, BMI, education, occupation, physical activity, PIR, health insurance, and smoking. Models with additional adjustments for estimated leukocyte proportions or without adjustments for self-rated health status are noted

*P* < 0.007: Bonferroni significant

*P* < 0.05: marginally significant and highlighted in bold italic

### Directly measured telomere relationships

Measured telomere length was moderately correlated with DNAmTL (r = 0.36, *P* < 0.001). Having ≥ 10 visits in the past year was not associated with directly measured telomere levels (β = 0.01, 95% CI − 0.09, 0.10, *P* = 0.89). In models including leukocytes, being hospitalized overnight in the past year was not associated with directly measured telomere levels (β = − 0.03, 95% CI − 0.13, 0.08, *P* = 0.54).

### Relationships of health status, access to care, and utilization with chronological age

For comparison, we tested associations with chronological age and these relationships were largely null (Table S7).

## Discussion

In this analysis of a nationally representative cross-sectional sample of U.S. adults aged 50–84 years, we examined relationships of self-rated health status, healthcare access, and healthcare utilization with epigenetic aging. After adjusting for chronological age, lifestyle factors, and health variables, we found statistically significant associations of self-rated health status with three measures of epigenetic aging that are strong predictors of morbidity and mortality (PhenoAge, GrimAge2, and DunedinPoAm). Furthermore, we identified DNA methylation predicted cigarette packyears, B2M levels, and CRP levels as factors that may explain the association of health status with GrimAge2. Independent of health status, health insurance, lifestyle factors, and health behaviors, we found largely null relationships of healthcare access and utilization with epigenetic aging. Reporting having a routine place where one receives healthcare was marginally associated with having a lower GrimAge2 while having ≥ 10 health visits in the last year and being hospitalized in the last year were marginally associated with having a shorter DNAmTL. These same measures of healthcare utilization were not associated with directly measured telomere length, suggesting that DNAmTL may be more sensitive to health status than directly measured telomere length. Comparative relationships of health status, healthcare access, and utilization with chronological age were largely null further suggesting that epigenetic aging biomarkers are more sensitive indicators of health processes.

Many studies have described relationships of epigenetic aging with objective measures of disease severity [19], but fewer studies have demonstrated that similar relationships exist with self-reported measures of health. One notable example is research in 1,175 participants of the United Kingdom’s Understanding Society Study (ages 29–95 years) that demonstrated associations of self-rated health with second and third generation clocks like DunedinPoAm and PhenoAge, but not first generation clocks like HannumAge and HorvathAge [3]. Similarly, work from 560 Australian ASPREE study participants (ages 70 years and older) reported statistically significant associations of PhenoAge, GrimAge2, and DunedinPACE, an updated version of the DunedinPoAm pace of aging measure used in our study but not available in NHANES, with self-rated health but only in women. Again, these authors observed no associations with first generation measures of HannumAge and HorvathAge. Moreover, the etiology of their observed sex difference was not obviously clear, but the authors postulated that it may be related to baseline age acceleration in males that is frequently attributed to hormonal and lifestyle/behavioral differences between males and females [18]. A different study of 1,059 Australians in the Melbourne Collaborative Cohort (mean age of 69 years) identified significant effect modification of the associations of GrimAge and DunedinPACE epigenetic age measures with mortality by self-rated health. Specifically, the authors observed greater hazard ratios in participants with fair-poor health [17]. Our observations of lower epigenetic aging (particularly with second and third generation measures) in participants with good–excellent health agree with this prior literature and with the broader paradigm of better health being associated with lower epigenetic aging. It is worth noting that these relationships are likely more robust with second and third generation clocks, because these latter clocks are trained on clinical measures [3, 4, 6]. Because DNA methylation estimates of some of these clinical variables were available, we were able to identify estimated lower cigarette packyears (a measure of smoking) and lower levels of B2M (involved in immune function), CRP (a non-specific marker of inflammation), and leptin (a hormone produced by fat tissue) as GrimAge2 components associated with good–excellent self-reported health status [32–34].

It has been hypothesized that epigenetic age measures may one day play a role in the clinical management of patients [8], but there is very sparse literature examining epigenetic aging relationships with measures of healthcare access and utilization that are important for shaping health status and clinical care. To the best of our knowledge, the present study addresses this research gap by reporting these relationships for the first time. Still, we observed largely null relationships of healthcare access and utilization with epigenetic aging. We suspected that this was due to health status, which we show as independently associated with epigenetic aging, being a primary driver of healthcare access and utilization. Given that measures of healthcare access and utilization were associated with self-rated health status in our study sample, we ran access and utilization models both including and not including health status as a covariate. Understanding the impact of health insurance on health, access, and utilization [35], we did the same for health insurance as a covariate. Still, our results were largely unchanged across these models for reasons that remain unclear. One possibility is the cross-sectional nature of our study as utilization and access relationships may be more apparent in longitudinal data. We attempted to explore longer-term relationships in our limited data, but observed no significant findings with the variable that considered changes in participants’ health in the last year. Still, we believe that there is some connection of self-rated health status with access and utilization based on our observation of a marginal association of having a routine place for healthcare with lower GrimAge2 levels. CRP and leptin are the GrimAge2 components marginally associated with this measure of healthcare access, and they were previously identified as components associated with self-rated health status. Future studies with truly longitudinal data will be useful for better characterizing the relationships of epigenetic age with healthcare access and utilization.

With specific attention to healthcare utilization, we observed marginal associations of having ≥ 10 healthcare visits in the last year and being hospitalized overnight in the last year with shorter DNAmTL. Consistent with prior studies [36, 37], we observed differences in the associations detected by DNAmTL and directly measured telomere length, further supporting the notion that DNAmTL may be more sensitive to health status. Most compellingly, an independent NHANES analysis revealed that DNAmTL has a stronger association with cardiovascular disease and long-term mortality than measured telomere length [28]. Additional evidence that these two measures are related but different comes from our study, where DNAmTL showed only a moderate correlation with measured telomere length—consistent with correlations reported in the original manuscript introducing DNAmTL [5]. While some theories suggest DNAmTL may better reflect processes like telomere maintenance mechanisms [23] and/or cell turnover and proliferation [5] rather than actual telomere length, the underlying mechanisms to explain the differences with measured telomere length remain an active area of research. Still, our findings are in agreement with the notion of shorter telomeres being associated with increased morbidity as individuals with more serious ailments are likely to have more doctor’s visits and be hospitalized [38]. More specifically, shorter directly measured telomere length has been associated with general hospitalization [39] and COVID-19 hospitalization [40] while shorter DNAmTL has been associated with hospitalizations in chronic obstructive pulmonary disease [41]. In our study, we did not have access to data describing why patients were hospitalized but future studies examining this question may reveal useful insights.

Our study has several strengths, including the use of DNA methylation-based biomarkers to directly assess relationships of health status, healthcare access, and healthcare utilization. However, certain limitations should be acknowledged. First, while NHANES includes a broad range of demographic and lifestyle variables, some data were missing for certain participants. To address this, we performed analyses using imputed covariates. Second, as our study is cross-sectional, it cannot capture longitudinal relationships, which are crucial for understanding many health processes, including chronic disease management and general health maintenance. There remains a need for future studies with longitudinal data to study these longitudinal relationships. Third, our dataset was limited in its measures of healthcare access and utilization. While our results are promising, they may not generalize to all dimensions of these constructs. Future studies could further explore the relationship of epigenetic aging with additional metrics of healthcare access (e.g., timeliness of care, transportation barriers, referral follow-up rates) and utilization (e.g., hospital length of stay, home healthcare use, rehabilitation service engagement). Fourth, the data used in our study are approximately 20 years old at the time of analysis, which may limit the generalizability of our findings given significant changes in the U.S. healthcare landscape over time. However, this remains the most recent methylation data available in NHANES. Despite these limitations, our study addresses novel questions, and the findings from this nationally representative sample can help guide future research using more recent data.

## Conclusions

In conclusion, among a representative sample of U.S. adults aged 50–84 years, we observed associations of self-rated health status with second and third generation epigenetic aging biomarkers (PhenoAge, GrimAge2, and DunedinPoAm). We also observed weaker relationships of healthcare access (having a routine place for receiving healthcare) with GrimAge2 and utilization (≥ 10 visits a year and being hospitalized overnight) with DNAmTL. If validated in future research, these findings suggest that epigenetic aging measures may be valuable for understanding healthcare access and utilization patterns that influence health outcomes. These findings also underscore the importance of accounting for healthcare access and utilization patterns in studies of epigenetic aging and disease processes, whenever possible and when not already addressed. Additionally, as health systems transition away from fee-for-service models (where health systems are paid for individual services/procedures) towards value-based care models (where payment is determined based on quality of care rather than quantity of services) epigenetic aging measures may be useful for monitoring the impact of this transition on patient health [42, 43].

## Data Availability

The datasets analyzed in the current study are available from the NHANES website.
